# Ultrasonographic findings, including small bowel intussusception, in acute food protein‐induced enterocolitis syndrome

**DOI:** 10.1111/pai.70036

**Published:** 2025-02-17

**Authors:** Yoshihiro Azuma, Yasufumi Sakata, Yuno Korenaga, Fumiko Okazaki, Hiroyuki Wakiguchi, Ken Fukuda, Shunji Hasegawa

**Affiliations:** ^1^ Department of Pediatrics Yamaguchi University Graduate School of Medicine Ube Japan

**Keywords:** food allergy, food protein‐induced enterocolitis syndrome, infant, intussusception, ultrasonography

To the editor

Food protein‐induced enterocolitis syndrome (FPIES) is a non‐IgE‐mediated food allergy affecting infants. Acute FPIES is characterized by severe, repetitive vomiting that begins 1–4 h after eating the food antigen, is accompanied by lethargy and pallor, and may be accompanied by watery diarrhea that usually develops 5–8 h later.^1^

The presentation of FPIES can mimic acute gastrointestinal diseases such as gastroenteritis. Therefore, physicians often find it difficult to diagnose FPIES. However, noninvasive and reliable tests for diagnosis in the acute phase have not yet been established.^2^

Ultrasonography (US) is recommended for evaluating patients with various pediatric gastrointestinal disorders.^3^ However, there have been few studies on ultrasonographic findings in non‐IgE‐mediated intestinal food allergies.^4^, ^5^ To our knowledge, no report has been published regarding ultrasonographic images of antigens other than cow's milk. Therefore, ultrasonographic features of FPIES remain unclear. In our hospital, US is performed in daily clinical practice for the diagnosis and management of pediatric gastrointestinal diseases including FPIES. In this study, we retrospectively investigated the ultrasonographic features of patients with FPIES who underwent OFC.

Patients aged <15 years who underwent OFC to confirm the diagnosis of FPIES or remission between May 2021 and July 2023 were included in this study. The patients were divided into two groups. The OFC‐positive group included patients who met the diagnostic criteria for the interpretation of OFCs, as described in the consensus guidelines.^1^ The OFC‐negative group included patients without gastrointestinal symptoms in the OFC. The patients who presented gastrointestinal symptoms during OFC but did not meet the diagnostic criteria were excluded. The OFC protocol was as follows: US was performed at two or three points: pre‐OFC, 6 h after the OFC for all patients, and 24 h after the OFC for patients in the OFC‐positive group. Blood samples were obtained pre‐OFC and 6 h after OFC. Fecal samples for occult blood tests were obtained pre‐OFC and 24 h after OFC. OFCs were performed in a single dose, and the dosage was determined according to the latest dose that induced gastrointestinal symptoms.

US was performed by experienced pediatricians with expertise in pediatric US using ARIETTA 850 (Fujifilm Healthcare, Chiba, Japan) with convex (1–5 MHz) and linear (2–12 MHz) transducers.

We evaluated intestinal ultrasonographic findings, including peristaltic movement, wall thickness, and fluid accumulation. Sonographers held the probe at the same area for at least 10 s, categorizing it as hypoperistalsis if no peristaltic movement was observed. Conversely, when continuous peristaltic movement was observed during probe fixation, it was categorized as hyperperistalsis. The measurement of the wall thickness of the gastrointestinal tract (stomach, duodenum, jejunum, ileum, and colon) and the assessment of fluid accumulation were based on previous studies.^6^, ^7^

Statistical analyses utilized JMP Pro version 16 (SAS Institute, Cary, NC, USA). Comparisons between groups employed Student's *t*‐test and Mann–Whitney *U* test, with significance set at *p* < .05.

Twenty‐five patients underwent OFC during the study period. Fourteen patients vomited during OFC. However, two patients were excluded because they did not meet two or more of the minor FPIES diagnostic criteria. Therefore, 12 patients were included in the OFC‐positive group and 11 patients were included in the OFC‐negative group. A total of 23 patients were included in the analysis. The patient characteristics are shown in Table 1. Of the 23 patients, 12 (6 in the OFC‐positive and 6 in the OFC‐negative group) met the diagnostic criteria for FPIES based on their medical history of symptoms. The median time from the last gastrointestinal symptoms to the OFC was 2.5 months (range: 0–24 months) in the OFC‐positive group and 7 months (3–16 months) in the OFC‐negative group. Antigen‐specific IgE (reference <0.7 UA/mL) was elevated in one patient in the OFC‐positive group (egg yolk 0.92 UA/mL) and in three patients in the OFC‐negative group (egg yolk 2.31 UA/mL, soybean 6.41 UA/mL, egg yolk 9.52 UA/mL).

**TABLE 1:** Patients' characteristics and ultrasonographic findings.

	OFC positive, *n* = 12	OFC negative, *n* = 11	*p*‐Value
Sex (M:F)	7:5	6:5	0.85
Age at OFC (month), median (range)	11 (6–35)	14 (10–31)	0.21
IgE (IU/mL), median (range)	20 (2–161)	17 (11–321)	0.70
Blood eosinophil count before OFC (/μL), median (range)	294 (60–540)	250 (60–1740)	0.98
Food protein			
Egg yolk	6	5	0.70
Soybean	2	6	0.08
Cow's milk	2	0	0.49
Wheat	2	0	0.49
Symptoms during OFC			
Vomiting	12	0	<0.001*
Diarrhea	5	0	0.023*
Lethargy	12	0	<0.001*
Pallor	4	0	0.056
Number of patients with neutrophil counts increased ≥ 1500 from baseline during OFC	10	3	0.012*
Fecal occult blood test 24 h after OFC (ng/mL), median (range)	201 (30–1000)	30 (30–221)	0.018*
Pre‐OFC ultrasonographic findings			
Small intestinal fluid accumulation	3	1	0.26
Small intestinal hyperperistalsis	0	2	1.00
6 h after OFC ultrasonographic findings			
Small intestinal fluid accumulation	10	1	0.0010*
Small intestinal hyperperistalsis	7	0	0.0028*
Small bowel intussusception	3	0	0.23

Abbreviation: OFC, oral food challenge.

* *p* < 0.05.

In the OFC‐positive group, jejunal wall thickness before OFC (median: range, 1.7:1.0–3.8 mm) significantly increased 6 h after OFC (3.2:1.6–5.0 mm). It significantly decreased 24 h after OFC (1.7:0.1–2.6 mm). The edematous jejunal wall thickening and fluid accumulation observed in this study are shown in Figure 1A,B, respectively. In the OFC‐negative group, no significant change was observed in the jejunal wall thickness between pre‐OFC (1.7:1.0–2.8 mm) and 6 h after OFC (1.6:1.0–3.6 mm). No significant changes were observed in the other parts of the intestinal tracts before or after OFC in either the OFC‐positive or OFC‐negative group (Table S1).

**FIGURE 1:**
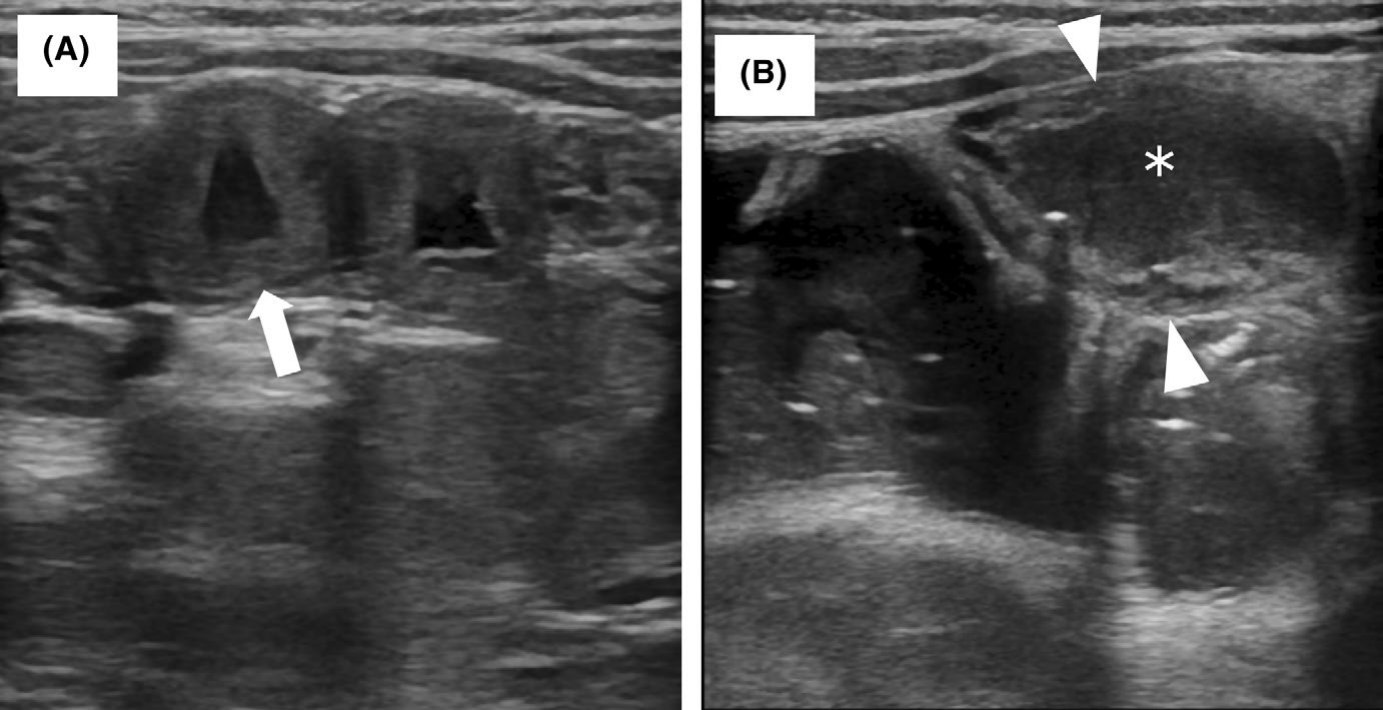
Ultrasonographic findings in the OFC positive group: (A) jejunal wall thickness with edema (arrow), measurement by placing one pointer at the mucosal layer and the other at the serosa in the same loop; (B) fluid accumulation depicted as a hypoechoic lesion (*) with bowel dilatation in the ileum (arrowhead). OFC, oral food challenge.

There were no significant differences in intestinal fluid accumulation or small intestinal peristalsis between the OFC‐positive and OFC‐negative groups pre‐OFC. In contrast, the number of patients with small intestinal fluid accumulation and hyperperistalsis was significantly higher in the OFC‐positive group than in the OFC‐negative group 6 h after OFC.

Three patients in the OFC‐positive group presented with small bowel intussusception 6 h after OFC. US revealed a bowel‐within‐bowel appearance, identified on transverse scans as a “doughnut sign” with multiple concentric rings (Figure 2A‐1,B‐1,C‐1), and on longitudinal scans as a “sandwich sign” with a fork appearance (Figure 2A‐2,B‐2,C‐2). The patients included one male (7‐month‐old, antigen: egg yolk) and two females (33‐month‐old, antigen: wheat; 24‐month‐old, antigen: wheat). The small bowel intestinal intussusception in two patients was observed in the left upper abdomen, which would lead us to suspect that it was located in the jejunum. In the remaining patient, the lesion was observed in the left lower abdomen, and therefore it was difficult to determine whether it was located in the jejunum or ileum. All three patients presented with recurrent vomiting, diarrhea, and lethargy. Small bowel hyperperistalsis and small intestinal wall thickening were also observed. The intussusceptions resolved spontaneously without intervention such as surgery or enema reduction. US performed 24 h after OFC showed improvement in intestinal wall thickness and hyperperistalsis. The fecal occult blood test results 24 h after OFC were >1000 ng/mL in the three patients.

**FIGURE 2:**
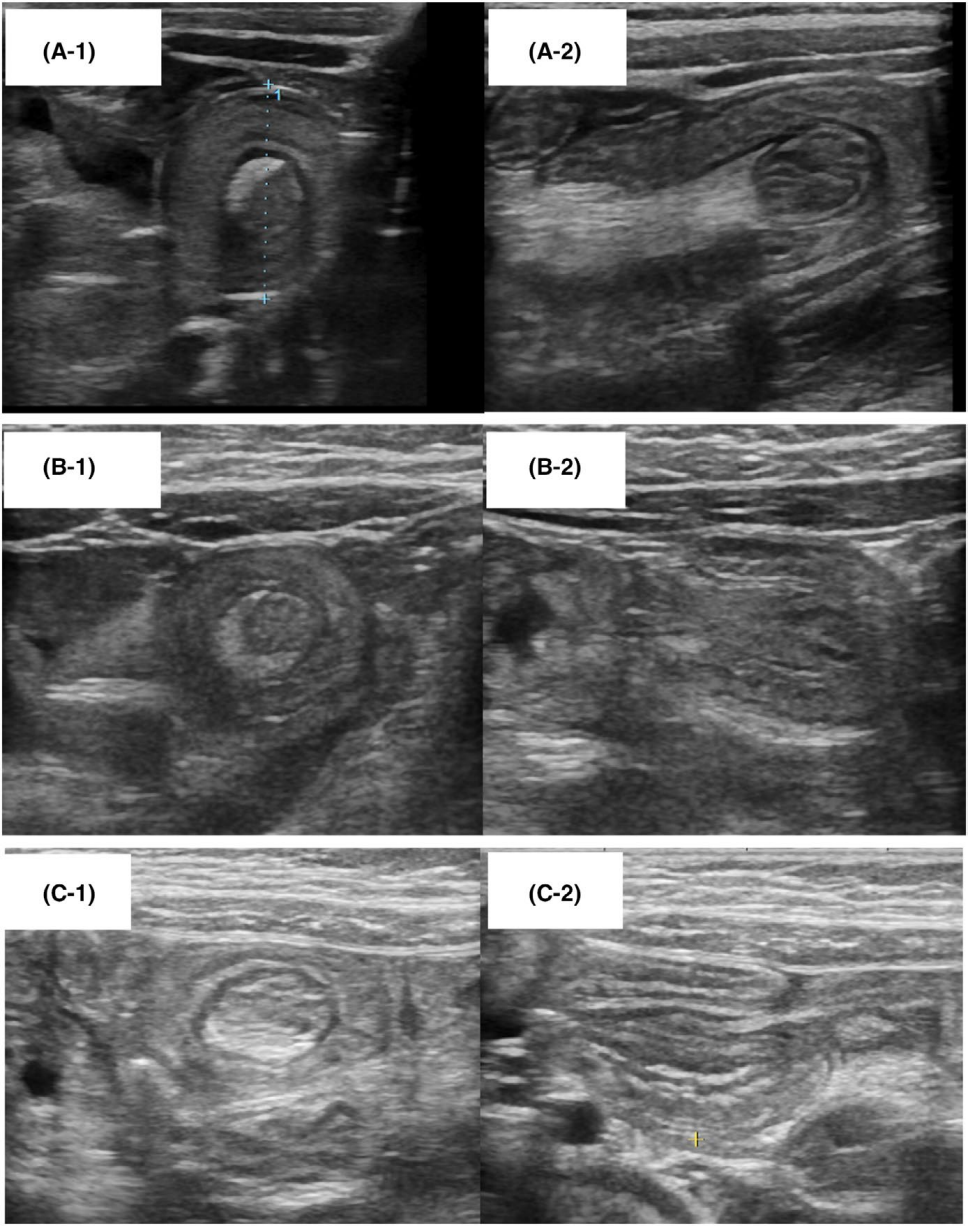
Ultrasound images of small intestinal intussusception in three OFC‐positive patients with FPIES. Observed on transverse scans as “donut signs” with multiple concentric rings, giving the target appearance, and on longitudinal scans as “sandwich sign” with a fork appearance. (A‐1) transverse scan of a 7‐month‐old boy; (A‐2) longitudinal scan of a 7‐month‐old boy; (B‐1) transverse scan of a 33‐month‐old girl; (B‐2) longitudinal scan of a 33‐month‐old girl; (C‐1) transverse scan of a 24‐month‐old girl; (C‐2) longitudinal scan of a 24‐month‐old girl.

In this study, we examined the characteristics of the ultrasonographic findings in patients with FPIES induced by several antigens.

The OFC‐positive group showed thickening of the jejunal wall and a higher rate of fluid accumulation in the small bowel and hyperperistalsis than the OFC‐negative group. Previous studies have reported that patients with non‐IgE‐mediated cow's milk allergy exhibited small intestinal thickening and fluid‐filled dilation.^4^, ^5^ Our findings align with previous research. Fluid accumulation in the small intestine has also been observed in patients with rotavirus gastroenteritis.^7^ Therefore, distinguishing acute FPIES from viral gastroenteritis based on fluid accumulation alone may be difficult. However, Yamashita et al. have reported that the thickness of the small intestinal wall of patients with rotavirus gastroenteritis did not change immediately after onset and over the course of the illness.^7^ In contrast, in patients in the OFC‐positive group, the thickening of the jejunal wall was observed 6 h after and had improved 24 h later. These findings suggest that a thickened jejunal wall, which improves in a short period, may be useful for the diagnosis of acute FPIES.

Hyperperistalsis was also observed in this study. However, Jimbo et al. reported decreased small intestinal peristalsis in patients in the US with non‐IgE‐mediated gastrointestinal cow's milk allergy.^5^ This discrepancy may be attributed to the lack of standardized techniques for evaluating peristalsis. Therefore, peristalsis may be evaluated differently, depending on the sonographer or part of the intestine.

Notably, small intestinal intussusception occurred in three patients with FPIES. Intussusception is a common cause of small‐bowel obstruction in infants. However, few studies have described the relationship between food allergies and intussusception. A prospective study of patients with intussusception suggested that food allergy, including non‐IgE‐mediated allergy, is a risk factor for recurrent intussusception.^8^ Although previous studies have suggested that food allergy can lead to intussusception, to the best of our knowledge, this is the first study that demonstrates that FPIES causes small intestinal intussusception. The etiology of small intestinal intussusception remains unknown. A case series of postoperative small intestinal intussusception suggests that local spasm or edema of the small intestine is the most plausible explanation for intussusception.^9^ In the present study, edematous intestinal wall thickening and hyperperistalsis were also observed in patients with small intestinal intussusception. We believe that these intestinal changes led to small intestinal intussusception in patients with FPIES.

All three patients with small intestinal intussusception recovered spontaneously without any invasive intervention despite suspected symptoms of intestinal obstruction. This is considered to be due to the improvement in gastrointestinal symptoms within a few hours in FPIES. Although most patients with small bowel intussusception are reported to be transient, a retrospective study reported that 62 patients with small intestinal intussusception planned surgery because of signs of obstruction at initial diagnosis.^10^ These findings suggest that FPIES should be considered a differential diagnosis in cases of small intestinal intussusception. Careful observation is necessary to determine the indications for surgery.

The limitations of this study include its retrospective and single‐center design. Ultrasound parameters such as peristalsis depend on the sonographer's subjective judgment. Prospective studies with standardized ultrasonographic protocols are necessary to validate this hypothesis.

Jejunal wall thickening, fluid accumulation, abnormal peristalsis, and small intestinal intussusception observed on ultrasound highlight its potential diagnostic value for acute FPIES. When small intestinal intussusception is present, FPIES should be considered a differential diagnosis.

## AUTHOR CONTRIBUTIONS

**Yoshihiro Azuma:** Conceptualization; methodology; data curation; investigation; formal analysis; visualization; project administration; writing – original draft; writing – review and editing. **Yasufumi Sakata:** Conceptualization; methodology; data curation; investigation; validation; writing – original draft; writing – review and editing. **Yuno Korenaga:** Conceptualization; data curation; methodology. **Fumiko Okazaki:** Conceptualization; methodology; data curation. **Hiroyuki Wakiguchi:** Conceptualization; methodology; supervision; data curation; writing – review and editing. **Ken Fukuda:** Funding acquisition; conceptualization; methodology; supervision. **Shunji Hasegawa:** Conceptualization; methodology; investigation; project administration; writing – review and editing; supervision; funding acquisition; formal analysis.

## FUNDING INFORMATION

This work was supported by JSPS KAKENHI (Grant number JP18K07848), a grant from the Kawano Masanori Memorial Public Interest Incorporated Foundation for Promotion of Pediatrics (Grant number, 27‐13), the Morinaga Foundation for Health & Nutrition, and a Grant‐in‐Aid for Translational Research from Yamaguchi University Hospital, 2018.

## CONFLICT OF INTEREST STATEMENT

The authors declare no conflicts of interest.

### PEER REVIEW

The peer review history for this article is available at https://www.webofscience.com/api/gateway/wos/peer‐review/10.1111/pai.70036.

## ETHICS STATEMENT

This study was approved by the Institutional Review Board of Yamaguchi University Hospital (H2023‐119). This was a retrospective study. Patients, parents, or guardians were not required to provide informed consent for the study because the analysis used anonymous clinical data obtained after each patient agreed to the OFC, including ultrasonography, by providing written consent. We also applied the opt‐out method using a poster on our hospital website to obtain consent for this study. The poster was approved by the Institutional Review Board of Yamaguchi University Hospital.

## Supporting information


Table S1.
