# Supplementary material for: Ultrasonographic findings, including small bowel intussusception, in acute food protein‐induced enterocolitis syndrome
Source: Pediatr Allergy Immunol. 2025 Feb 17;36(2):e70036. doi: 10.1111/pai.70036 (PMC11831709; doi:10.1111/pai.70036)
Supplement: Supplementary file 1 — Table S1. [file PAI-36-e70036-s001.docx]

|  | **OFC-positive** | | | **OFC-negative** | | |
| --- | --- | --- | --- | --- | --- | --- |
|  | Pre-OFC | 6h after OFC | p-value | Pre-OFC | 6h after OFC | p-value |
| stomach (mm)  median (range) | 1.6  (0.9-3.8) | 2.1  (0.9-4.2) | 0.22 | 1.5  (1.2-3.2) | 1.6  (0.7-2.6) | 0.54 |
| duodenum (mm)  median (range) | 1.2  (1.0-1.9) | 1.1  (0.9-3.4) | 0.37 | 1.7  (1.4-2.6) | 1.4  (0.7-2.3) | 0.11 |
| jejunum (mm)  median (range) | 1.7  (1.0-3.8) | 3.2  (1.6-5.0) | *0.017 | 1.7  (1.0-2.8) | 1.6  (1.0-3.6) | 0.53 |
| ileum (mm)  median (range) | 1.5  (0.6-2.7) | 1.8  (0.2-4.4) | 0.11 | 1.3  (0.7-2.1) | 1.3  (0.7-3.8) | 0.30 |
| ascending colon (mm)  median (range) | 0.9  (0.6-1.7) | 1.1  (0.1-3.9) | 0.73 | 1.1  (0.7-1.8) | 1.2  (0.7-1.5) | 0.95 |
| transverse colon (mm)  median (range) | 1.1  (0.6-1.8) | 0.8  (0.1-2.0) | 0.55 | 0.9  (0.6-1.4) | 1.0  (0.7-1.8) | 0.55 |
| descending colon (mm)  median (range) | 1.2  (0.6-1.9) | 1.2  (0.2-4.3) | 0.15 | 1.0  (0.7-1.6) | 1.1  (0.9-3.1) | 0.12 |
| sigmoid colon(mm)  median (range) | 1.0  (0.1-1.9) | 1.3  (0.1-2.6) | 0.16 | 1.0  (0.8-1.7) | 1.2  (0.1-2.4) | 0.68 |

**Supplementally table 1: intestinal wall thicknesses in OFC-positive and OFC-negative groups**

OFC: oral food challenge ＊＜0.05
